# Gender issues in nursing research

**DOI:** 10.4069/kjwhn.2022.09.19

**Published:** 2022-09-30

**Authors:** Cheol-Heui Yun

**Affiliations:** Department of Agricultural Biotechnology, and Research Institute of Agriculture and Life Sciences, Seoul National University, Seoul, Korea

## Introduction

Sex and gender-based analysis (SGBA) is a recommended framework and methodology that enhances critical accuracy in research. Scientific research aims to create objective and universally valid knowledge that transcends cultural constraints in terms of methods, experimental techniques, and epistemology. However, when it comes to gender, race, and other social concepts, science is not value-neutral. When taking a scientific approach, the act of identifying correlations between scientific concepts and gender-related social structures can expand the scope of typical critical research pursued in scientific studies. SGBA begins by including women in studies of human subjects as well as evaluating sex and gender differences in basic scientific research. Health status and treatment outcomes related to biological (sex) and sociocultural (gender) differences will not only improve our understanding of the health and well-being of men and women but also enhance our healthcare environment and direction of future research.

In medical research, sex and gender biases and imbalances are typically caused by flawed sampling methods when designing and conducting experiments. It is well-known that the results of studies involving only men are often assumed to apply equally to women under the premise that women are equivalent to men. The inconvenient truth, however, is that studies that consider both men and women or only women are often considered impractical, too expensive, and/or potentially unsafe. Consideration of women’s hormonal cycles is often thought to complicate research design and analysis and result in unnecessary research expenses, constituting a methodological problem [1]. As such, the results of studies on men (diagnosis, preventive measures, and treatment) are often erroneously applied to women. As a result, drug side effects occur 1.5 to 2 times more frequently in women. If a different sampling method that considers sex and/or gender were applied, problems due to bias in existing medical and health research could be reduced. Therefore, there is an increasing demand for the development of a standard method that entails the inclusion of an appropriate proportion of women and minorities.

Sex and gender are recognized as important determinants of health and well-being and both should be thought of as ethically important considerations in nursing research. In addition to the simple biological sex-based distinction between male and female, researchers should be mindful that gender encompasses the roles, behaviors, and identities of various members of society, and gender issues influence how people interact and perceive each other [2].

As such, gender issues are highly significant since they open up a diverse and extensive research environment related to nursing in terms of the social, cultural, political, and economic aspects of research beyond the level of individual patients or medical consumers. Therefore, gender-related research will help nursing researchers understand the broader societal context. Gender research can also help identify and resolve social inequality in nursing research and healthcare and address the needs of marginalized groups and minorities in terms of medical rights [3].

Gender research is considered a very important factor for expanding possibilities to improve nursing practice, health outcomes, and, more broadly, social and political changes to achieve social justice in the healthcare system. The intersection of nursing and gender research is suggested as a strong pairing that can challenge current sex and gender-related imbalances and highlight important questions. In addition, nursing research must change alongside gender research and address social and cultural questions related to gender issues.

## Sex and gender equity in nursing research

The field of nursing, which was mainly developed by women, unfortunately appears to have been considered at a low status within academia. However, recent efforts to fully recognize the role of gender within nursing have led to the following impacts on the overall development of nursing education and research [4].

### Gender research helps us understand the sociopolitical context of nursing research

Gender research is an important factor that considers social, cultural, political, and economic differences related to race, gender, and class. Gender research in nursing provides an analytic method for understanding the structure of healthcare and social and cultural contexts, thereby illuminating many issues, including discriminatory practices, inequality, and cultural differences. Gender research can expand the knowledge base of nursing education and determine future directions in professional nursing research. Adopting a feminist approach in nursing is a meaningful attempt to expand the body of nursing knowledge and improve the quality of nursing services by revising the existing, male-oriented knowledge base and providing a vision for empowering women. Furthermore, in nursing, the feminist approach presumably contributes to the development of nursing knowledge by combining feminist research and qualitative research. Nursing knowledge will ultimately play an important role in providing an interactive relationship that goes beyond “male-centered” and “human-centered” by introducing feminism in the field extended by subjective experience derived from a qualitative approach [5].

### Gender research does not just advance traditional nursing research, but also pioneers new fields

Nursing research has carved out its contributions to improving patient care and adding to the knowledge base of health research. Adapting a gender research framework will encourage collaboration between nursing and researchers in other fields to identify gaps in current research and advance new research areas through mutual collaboration.

### Gender research should exist as a form of critique within nursing research

Nursing research that fails to consider gender research lacks perspective in terms of gender, as seen in many existing studies across various fields that fall short of considering gender bias or gender sensitivity; thus, limited to ‘half the picture.’ To identify limitations in existing nursing research and move toward change and innovation, it is essential to incorporate gender research into nursing research.

The lack of SGBA, including the insufficient proportion of female study participants, poses a barrier to our understanding of health. For example, while about half of coronary heart disease (CHD) patients are women, women only comprised around 25% of participants in CHD trials [6]. To address these issues, it is important to consider (1) factors such as age, socioeconomic status, race, and ethnicity in research and reports; as well as (2) sex and gender and their interactions [7]. Research ethics committees (RECs; any committee established by an organization or institution to review the ethical aspects of research with human being) can play a pivotal role in designing studies and identifying sex and gender gaps in the early stages of research protocol development. A study investigating whether RECs deliberate on sex and gender analysis in health-related research pointed out a lack of awareness of the importance of gender in research design and gender-related education offered by RECs [8].

The Canadian Institutes of Health Research introduced an SGBA policy in 2009, and the European Commission has called for an integrated approach to incorporating “gender dimension” in biomedical research for the analysis of sex and gender [9]. Given such international trends, the active response of nursing research encompassing SGBA would serve to expand the scope of research and spur its action.

## Sex and Gender Equity in Research guidelines

**Figure 1.**
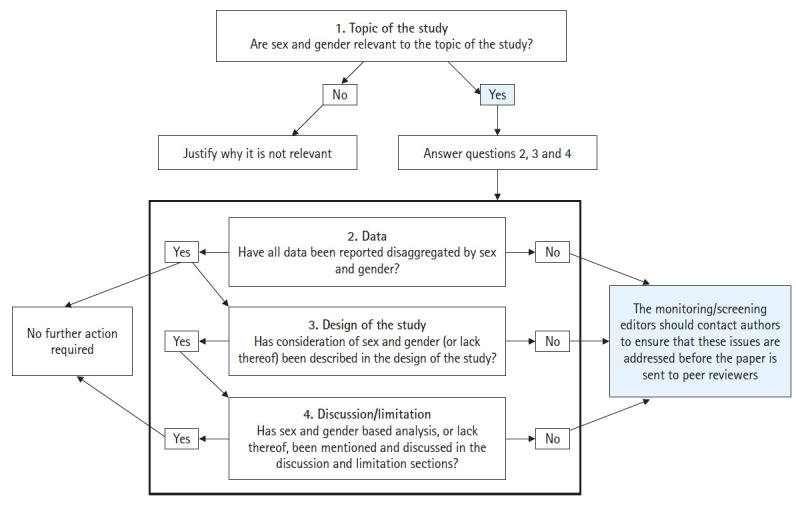
Sex and Gender Equity in Research (SAGER) flowchart guiding editors’ initial screening of submitted manuscripts. Adapted from Heidari et al. [10] according to the Creative Commons License.

The Sex and Gender Equity in Research (SAGER) guidelines play an important role in helping RECs strengthen health-related research methods from the conception of research protocols to publication. These recommendations provide guidance to journal editors (Figure 1 [10]), as well as authors and publishing professionals to ensure that sex and gender are considered and reported appropriately in journals [11]. The SAGER guidelines have also been translated into Korean and can be found on the European Association of Science Editors website (https://ease.org.uk/communities/gender-policy-committee/the-sager-guidelines/). The recommendations of the guidelines delineate what needs to be specified in reporting for the title and abstract, introduction, methods, results, and discussion sections. Meanwhile, potential issues can be divided into five categories: (1) concerns about mandating; (2) lack of time, capacity, and resources; (3) resistance or lack of awareness; (4) technical challenges; and (5) looking ahead.

### Concerns about mandating

According to the SAGER guidelines, gender-based analysis requires a different approach than usual, requiring additional time and expenses to use as large a sample as possible. Several research funding agencies have provided funding/grants for this type of analysis, which led to the establishment of the SAGER guidelines as a successful model.

### Lack of time, capacity, and resources

Journal editors may lack the time, ability, and resources to compel authors to comply with SAGER guidelines. In addition, journals with relatively few published works per year may find it very difficult to enforce SAGER guidelines. However, efforts should be made to implement these guidelines to improve the scientific quality of research papers.

### Resistance or lack of awareness

Some journals may refuse to comply with SAGER guidelines or find them inapplicable to their fields. All journals, however, should perform routine education and promotion for peer reviewers related to sex and gender. Online training courses developed by the Canadian Institutes of Health Research (training resources at https://cihr-irsc.gc.ca/e/50509.html; sex and gender training modules at https://www.cihr-irsc-igh-isfh.ca/) will help enhance the understanding of sex and gender inclusion in health research.

### Technical challenges

To minimize technical challenges, journal editors or publishers can implement checklists in their submission systems to integrate or customize the form for authors and peer reviewers to comply with SAGER guidelines.

### Looking ahead

The SAGER guidelines provide journals with an opportunity to improve their research and reporting practices. In the short-term, many journals have incorporated the SAGER guidelines into their author instructions, but a broader understanding and further efforts are needed to ensure better implementation of the SAGER guidelines. In addition, efforts to include age, race, ethnicity, social identity, and geographic diversity in the SAGER guidelines should continue to positively affect health and societal outcomes. Although it is important for publishers and journals to implement the SAGER guidelines, this should not just be their responsibility, but rather the responsibility of everyone involved in a published work, especially the authors.

## Conclusion

In conclusion, SGBA is a timely and significant issue for nursing research and the SAGER guidelines provide a frame of reference for adequate reporting of research on sex and gender. The *Korean Journal of Women Health Nursing* currently encourages accurate use of terms and reporting analysis of sex and/or gender, as appropriate (https://www.kjwhn.org/authors/authors.php). I hope more nursing journals and nurse researchers, especially those in Korea, actively adapt this framework and lead the way to making SGBA the norm, not the exception.
